# Supplementary material for: Airway environment drives the selection of quorum sensing mutants and promote Staphylococcus aureus chronic lifestyle
Source: Nat Commun. 2023 Dec 8;14:8135. doi: 10.1038/s41467-023-43863-2 (PMC10709412; doi:10.1038/s41467-023-43863-2)
Supplement: Supplementary file 1 — Supplementary information [file 41467_2023_43863_MOESM1_ESM.pdf]

Airway environment drives the selection of quorum sensing mutants and promote *Staphylococcus aureus* chronic lifestyle

Ding et al.

Figures S1 to S7

Tables S1 to S3

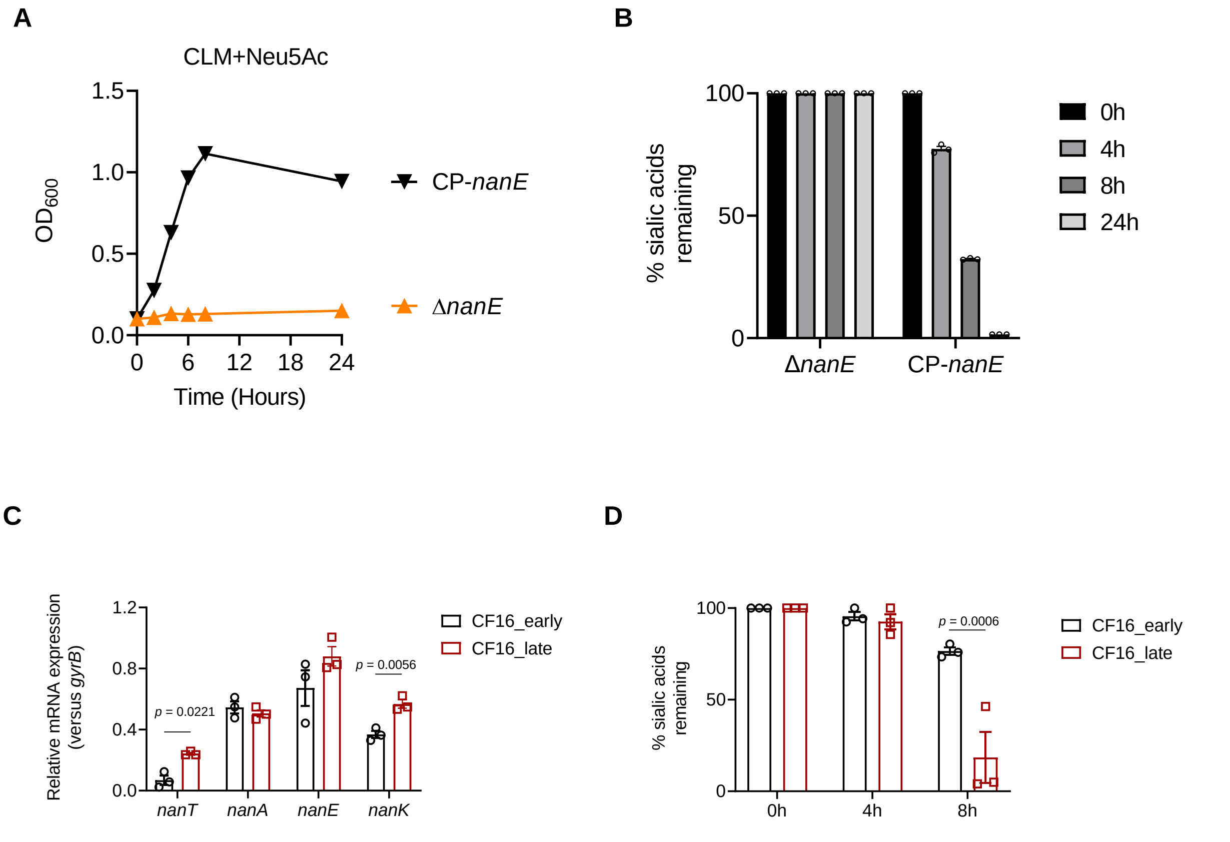

Fig. S1. Characterization of *nanE* deletion mutant and clinical isolates.
(A) Bacterial growth of Δ*nanE* mutant and complemented strain was monitored in CLM supplemented with 1.3 mM Neu5Ac (CLM+Neu5Ac).
(B) Kinetics of consumption of sialic acid (n = 3 samples/group) in Δ*nanE* mutant and complemented strain.
(C) The expression of *nan* locus from CF16 clinical isolates grown in CLM+Neu5Ac was quantified by qRT-PCR relative to that of housekeeping gene *gyrB* (OD600nm ~0.6) (n = 3 samples/group).
(D) Kinetics of consumption of sialic acids (n = 3 samples/group) in CF16 clinical isolate pair.
Error bars indicate mean with SEM. Statistically significant differences were calculated by one-way ANOVA with Bonferroni's multiple comparisons test and *p* value was indicated.

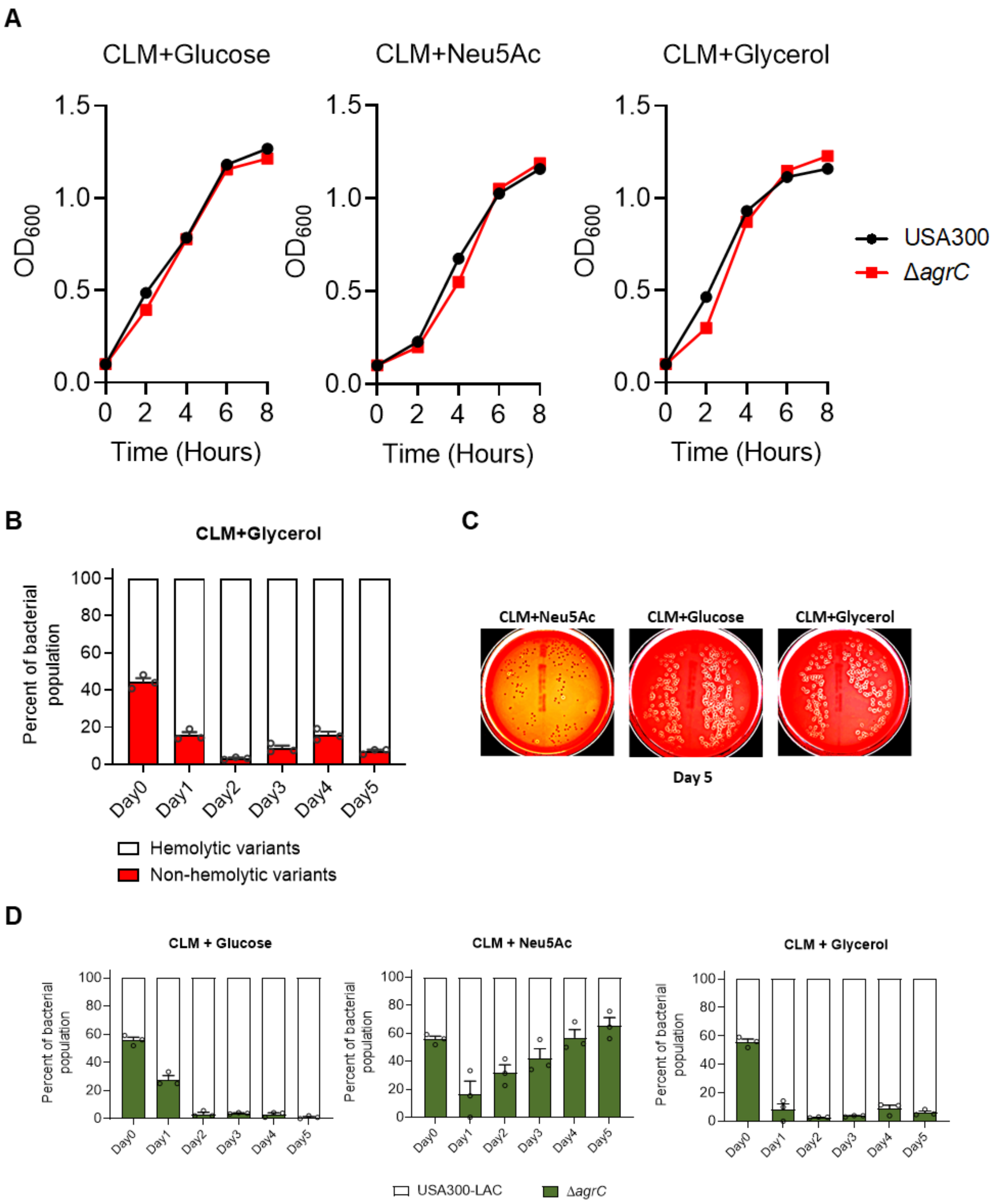

Fig. S2. Impact of carbon sources on proportion of Δ*agrC* mutant in competition assays.
(A) The growth of USA300-LAC or Δ*agrC* mutant in monoculture was monitored in CLM supplemented with 1.3mM glucose, Neu5Ac or glycerol. The results shown correspond to a representative experiment.
(B, C, D) Agr dysfunctional variant monitoring in competition assay. USA300-LAC (USA300) strain was competed for 5 days with Δ*agrC* derivative starting at a ratio of 1:1. The phenotype of USA300 and Δ*agrC* colonies is hemolytic and non-hemolytic, respectively.
(B) Competition assay in the presence of 1.3 mM glycerol. Proportions of nonhemolytic and hemolytic subpopulations were determined based on colony phenotype on sheep blood agar (n = 3 samples/group).
(C) Phenotypes of hemolytic and non-hemolytic bacterial subpopulations grown in CLM supplemented with 1.3mM glucose, Neu5Ac or glycerol on blood agar at day 5 of the competition assay.
(D) Percentage of USA300-LAC and Δ*agrC* mutant subpopulations was determined based on growth on erythromycin selective plates (n = 3 samples/group). Erythromycin susceptible colonies correspond to USA300-LAC and erythromycin resistant colonies correspond to Δ*agrC*.
Error bars indicate mean with SEM.

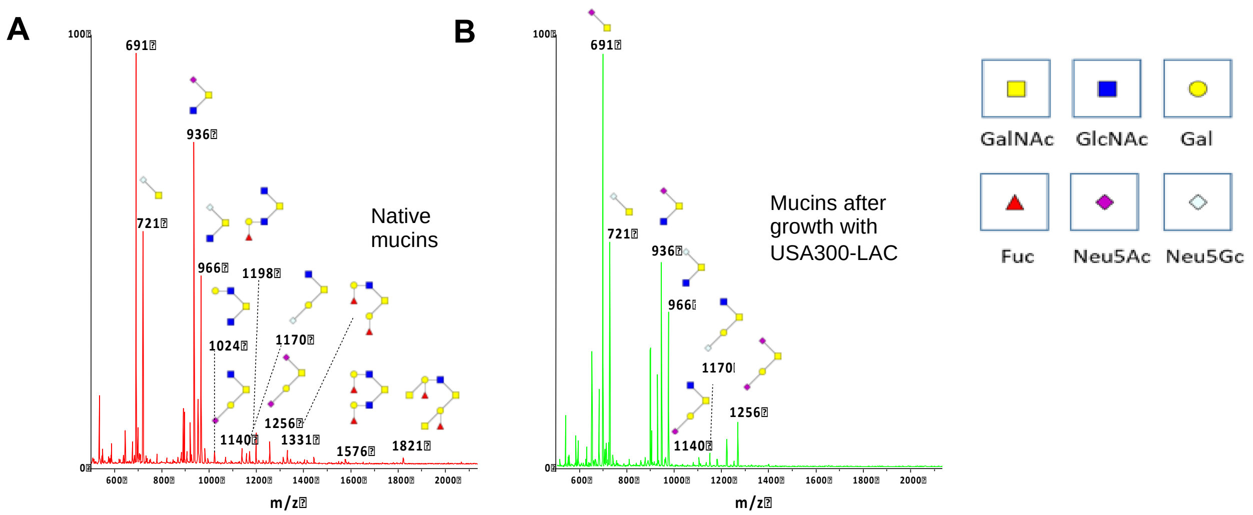

C

Proposed structure of blood group antigen carrying oligosaccharides	[M+Na]+	Native BSM	BSM after growth with USA300-LAC
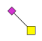	691	25.8+/-2.1%	25.3+/-1.8%
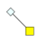	721	14.8+/-1.3%	9.8+/-0.9%
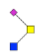	936	21+/-3.2%	30+/-4,5%
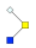	966	12.4+/-0.8%	13.8+/-1.9%
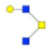	1024	1+/-0.1%	1.6+/-0.2%
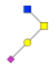	1140	1.1+/-0.2%	1.8+/-0.3%
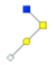	1170	1+/-0.3%	1.2+/-0.4%0
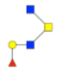	1198	2.1+/-0.5%	1.2+/-0.4%0
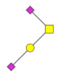	1256	1.5+/-0.2%	2.6+/-0.3%0
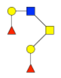	1331	1+/-0.2%	nd
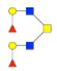	1576	1+/-0.2%	nd
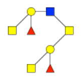	1821	0.6+/-0.1%	nd

Fig. S3. *S. aureus* lacks the ability to release sialic acid from mucin.
(A, B) MS spectra of permethylated O-glycans isolated from native bovine submaxillary mucins (BSM) (A) and MS spectra of permethylated O-glycans after growth with USA300-LAC (B). Mucin O-glycans were released from the protein backbone and permethylated before analysis by MALDI-TOF mass spectrometry in the positive ion mode [M+Na]+. Monosaccharide symbols according to the Consortium for Functional Glycomics (CFG) nomenclature. Key: fucose (red triangle), GlcNAc (blue square), sialic acid (purple diamond), galactose (yellow circle) and GalNAc-ol (yellow square). Note that for simplified comprehension, only the structure corresponding to the major isomer was drawn on the MS spectrum of BSM. Other isomers with the same monosaccharide composition might be present.
C) Major neutral and acidic oligosaccharide structures identified in native bovine submaxillary mucins (BSM) and BSM treated with USA300-LAC. Results are presented as mean±SD of percent of each oligosaccharide (nd, not detected).

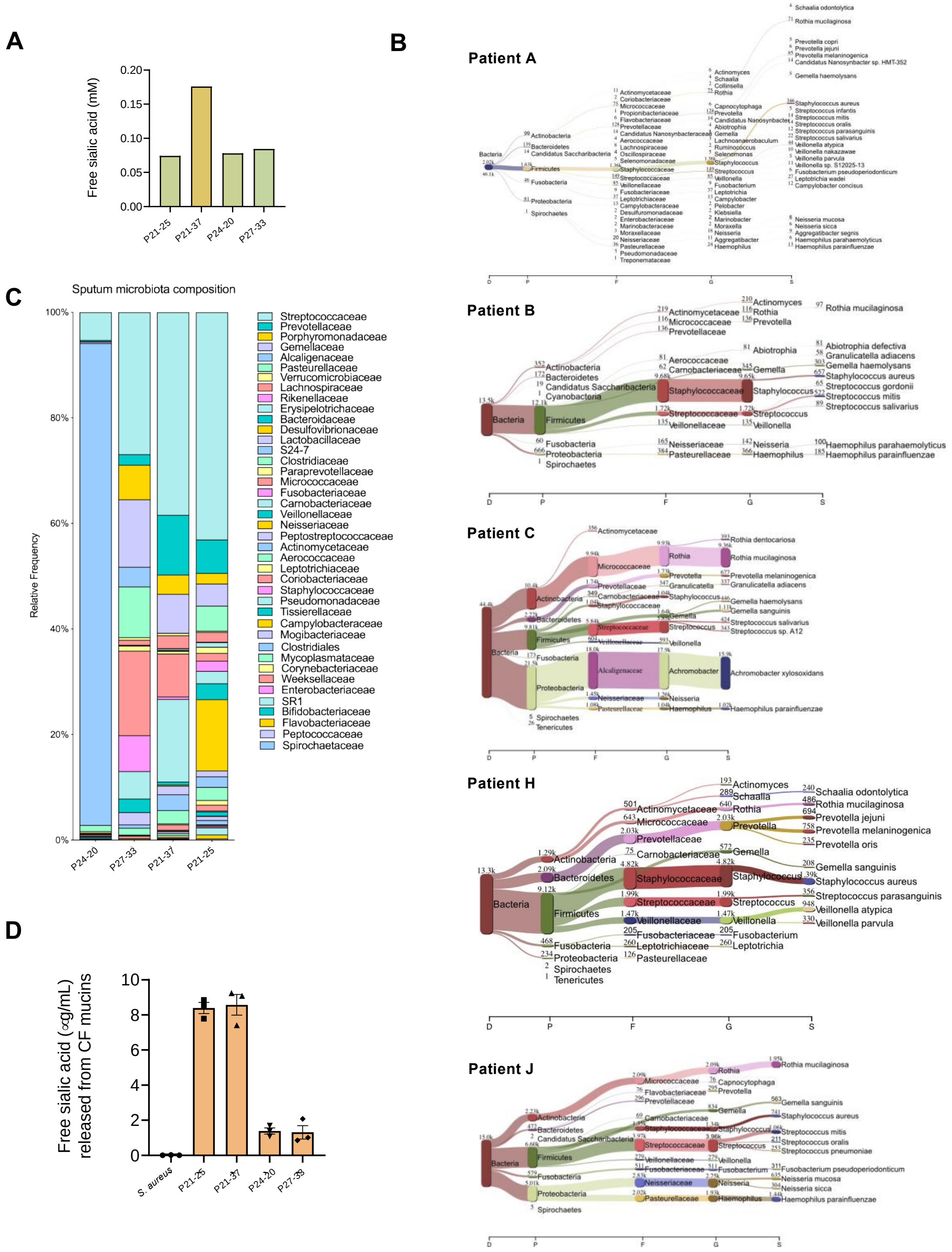

Fig. S4. Free sialic acid quantification and microbiota composition in sputa from CF patients.

(A) The concentration of free sialic acid was measured by colorimetric assay in the sputa obtained from four children with CF who did not have a *S. aureus* infection by standard microbiology culture.

(B) Shotgun metagenomic sequencing analysis of microbiota of five patients with *S. aureus* infection in standard microbiology culture.

(C) Taxonomic composition based on 16S analysis of microbiota in four CF patients' sputa without *S. aureus* by standard microbiology culture.

(D) The sialidase activity of four *S. oralis* isolates obtained in culture from sputa of the four patients without *S. aureus* was evaluated by quantifying the amount of salic acid released from purified CF patient mucins. The USA300-LAC strain was used as a negative control (n = 3 samples/group). Error bars indicate mean with SEM.

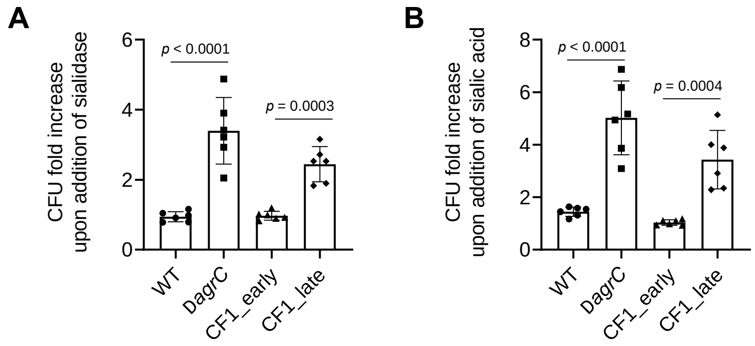

Fig. S5. Infection of ALI model with cystic fibrosis bronchial epithelial cell line (F508del CFBE41o-).
(A, B) ALI mucus of wells was apically infected with USA300, Δ*agrC*, or early and late isolates from patient CF1. Mucus was collected 16 hours post infection and plated for CFU counting. Data are represented as fold increases over wells infected with the same strains without sialidase/sialic acid addition (n=3 wells/groups). Error bars indicate mean with SEM. Statistically significant differences were calculated by one-way ANOVA with Bonferroni's multiple comparisons test and *p* value was indicated.
(A) 5 µL sialidase (0.5 U/mL DPBS/0.1% BSA) or 5 µL DPBS/0.1% BSA was added 24 hours before infection.
(B) 5 µL Neu5Ac (0.4g/L) or 5 µL H2O was added 2 hours before infection.

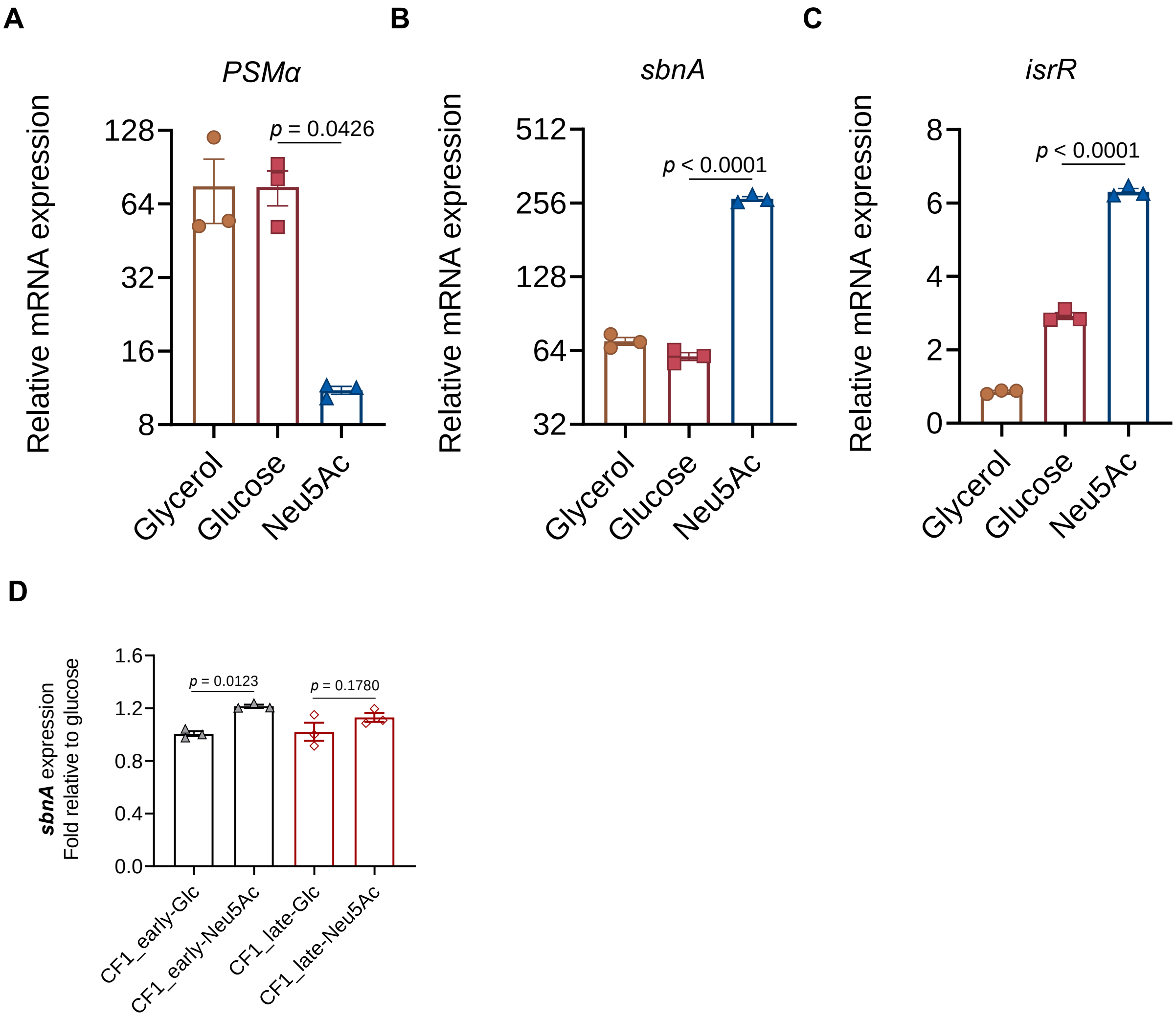

Fig. S6. Neu5Ac downregulates virulence factors but upregulates *isrR* and *sbn* genes.
(A) *PSM-α* (B) *sbnA* and (C) *isrR* expression from bacteria grown in CLM supplemented with glycerol, glucose or Neu5Ac (1.3 mM) were quantified by qRT-PCR with reference to *gyrB* in 3 independent cultures (n=3 samples/groups). (D) CF1_early and late isolates were cultivated in CLM supplemented with glucose or Neu5Ac (1.3 mM). The *sbnA* gene expression (OD600nm ~0.6) was quantified by qRT-PCR with reference to *gyrB*. Fold change was calculated relative to the glucose condition (n=3 samples/groups).
Error bars indicate mean with SEM. Statistically significant differences were calculated by one-way ANOVA with Bonferroni's multiple comparisons test and *p* value was indicated.

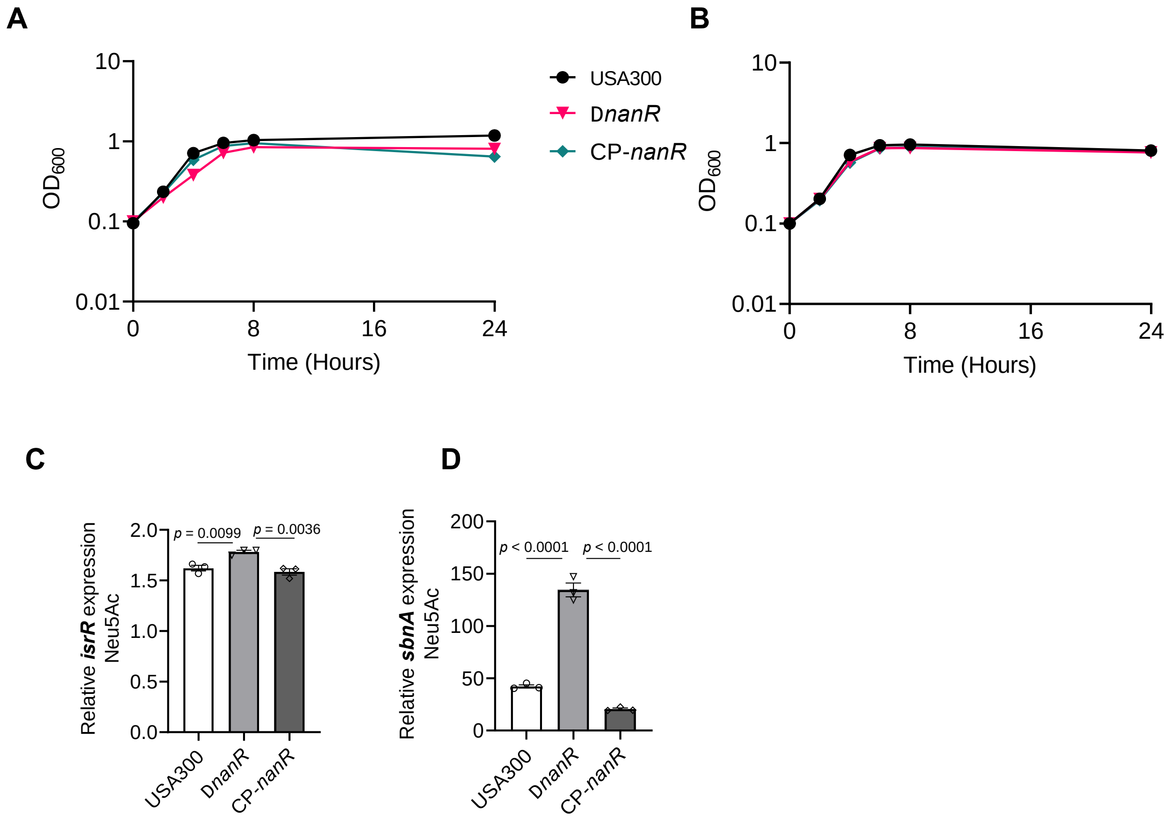

Fig. S7. Characterization of *nanR* deletion mutant and complemented strain.

(A, B) Bacterial growth of USA300, Δ*nanR* deletion mutant or complemented *nanR* strain (CP-*nanR*) was monitored in CLM without any additional carbon source (A) and, CLM supplemented with 1.3 mM Neu5Ac (B). The results shown correspond to a representative experiment.

(C, D) USA300, Δ*nanR* deletion mutant or complemented *nanR* strain (CP-*nanR*) was cultivated in CLM supplemented with Neu5Ac. *isrR* (C) and *sbnA* (D) gene expression (OD600nm ~0.6) were quantified by qRT-PCR with reference to *gyrB* (n = 3 samples/group).

Error bars indicate mean with SEM. Statistically significant differences were calculated by one-way ANOVA with Bonferroni's multiple comparisons test and *p* value was indicated.

Table S1. Strains and plasmids used in this study.

Strain or plasmid	Relevant characteristic(s)	Source of reference
**Strains**		
DH5α	Φ80’ *lacZ* ΔM15 *gyrA96 recA1 relA1 endA1 thi-1 hsdR17*	Invitrogen
USA300 LAC	MRSA containing phage ΦSA2usa (PVL), Ery^R	BEI Resources
USA300-Ery^S	Erythromycin-sensitive derivative of USA300	Laboratory collection
RN4220	Restriction-deficient transformation recipient *S. aureus*	Laboratory collection
USA300 Δ*nanR*	*nanR* defective mutant, Kan^R	This study
USA300 Δ*nanR*-CP-*nanR*	Complemented strain, USA300 Δ*nanR* containing pCN57-CP*nanR,* Ery^R Kan^R	This study
USA300 Δ*nanE*	*nanE* defective mutant, Kan^R	This study
USA300 Δ*nanE*-CP-*nanE*	Complemented strain, USA300 Δ*nanE* containing pCN57-CP*nanE,* Ery^R Kan^R	This study
S. mitis B26E10	referenced as 0902 230473 in the Necker Hospital collection	Laboratory collection
**Plasmids**		
pMAD	Plasmid with a thermosensitive origin, used for gene deletion; Amp^R Ery^R	Laboratory collection
pCN57	Plasmid containing *gfp* expression cassette (constitutive expression), Ery^R	Laboratory collection

Table S2. Primers used for RT-qPCR.

Gene name	Primer sequence (From 5' to 3')
gyrB	F: GCCATCCCAACTTAATAACCATGTAAAATTAGC
	R: GCGATGTTGTTTACGATAGCTTACATGCTAG
nanT	F: GGTCATAATGGTGCATCTCGTGC
	R: CATTGGATTTGTTTGAACCAGCAAGG
nanA	F: GGTGGATTTGAAAACTTAGAGGACGAAGAC
	R: CATACCTTGAACACCACGACCACC
nanE	F: GGTGCCATAAATAGATTCGATATGTCCTT
	R: CTTTTGATTTCACCGATTACAGTAGGTACT
nanK	F: CGCGTGAAGATGTGTTCAAGAC
	R: CTATCTCTTTATCGTGAACTTGAAG
nanR	F: GAAGATTTTATCGAAATGCGC
	R: CATCATATGTAGCACCAGCTCTC
sbnA	F: GAAGTGCATCCAAACGCACA
	R: CGACTAGCACCGATACCAGG
PSM-α	F: TATCAAAAGCTTAATCGAACAATTC
	R: CCCCTTCAAATAAGATGTTCATATC
isrR	F: ACAACGTTTCGTTCTTGTTGGA
	R: AGTGTCGTAAGGGTTTACTGCT

Table S3. Primers used for deletion mutant construction and complementation.

**Primer name**	**Primer sequence (From 5' to 3')**
seq-pMAD	F: TCTGGCCATTGCTCTGGGTTA
	R: GCTGTCCCTGATGGTCGTCAT
seq- pCN57	F: GCTCACATGTTCTTTCCTGCGTTATC
	R: CGCGAAAGTAGTGACAAGTGTTGG
pMAD-nanR	F: gGATCCGATATCGCCCGACG
	R: GAATTCGAGCTCCCGGGTA
nanR-up	F: gtacccgggagctcgaattcTAATTAGGTGCCTTTAATTAATGATG
	R: atccatacaaCGTTGAACACGATTCTCAAATTTC
nanR-KM	F: gtgttcaacgTTGTATGGATTAGTCGAGC
	R: ttgtctaggaTCAGAAGAACTCGTCAAG
nanR-down	F: gttcttctgaTCCTAGACAACAAATAACTAG
	R: cgtcgggcgatatcggatccATGTCAAAGTAAAAAATGATGTAAAC
pCN57-nanR	F: GCGTATGAAGGTGGTGCTGTgcggtaatacggttatccac
	R: CCACCTGCAACTGTAGACAAgctgcatgcagcttactatg
CP-nanR	F: gtggataaccgtattaccgcACAGCACCACCTTCATACGC
	R: catagtaagctgcatgcagcTTGTCTACAGTTGCAGGTGG
seq-nanR	F: ACGCGTGGCGTTGTCG
	R: AGGTATTTCATCAGCAGGC
pMAD-nanE	F: GAATTAATTCTCATGTTTGACAGCTTATC
	R: TTGAAGACGAAAGGGCCTC
nanE-up	F: cgaggccctttcgtcttcaaGAGTGTCGCAATTTGTGTG
	R: atccatacaaGTAGTGCCTGACAAGATAC
nanE-KM	F: caggcactacTTGTATGGATTAGTCGAGC
	R: accaccaacgTCAGAAGAACTCGTCAAG
nanE-down	F: gttcttctgaCGTTGGTGGTGCGATAAC
	R: tcaaacatgagaattaattcTGCATGGCTACCGCCAATC
pCN57-nanE	F: GTTCAAATTATGGAAGATTAAGATCCCCGGGTACCGAGCT
	R: CCTCACTCCATAAGTCGTGTCGACCTGCAGAATAAACCC
CP-nanE	F: GGGTTTATTCTGCAGGTCGACACGACTTATGGAGTGAGG
	R: AGCTCGGTACCCGGGGATCTTAATCTTCCATAATTTGAAC
seq-nanE	F: CTCGTTGGGACAATCACG
	R: GCCACGATTATTGCGACTGG
