# Supplementary material for: Airway environment drives the selection of quorum sensing mutants and promote Staphylococcus aureus chronic lifestyle
Source: Nat Commun. 2023 Dec 8;14:8135. doi: 10.1038/s41467-023-43863-2 (PMC10709412; doi:10.1038/s41467-023-43863-2)
Supplement: Supplementary file 3 — Description of Additional Supplementary Files [file 41467_2023_43863_MOESM3_ESM.pdf]

<u>Description of Additional Supplementary Files</u>

Supplementary Data 1: Identification of SNP within genomes of 10 hemolytic isolates and 10 non-hemolytic isolates at day 5 of 2 independent competition experiments compared to parental strain.

Supplementary Data 2: Analysis of RNA-seq data for the wild-type strain in the presence of glucose or sialic acid.
