# Supplementary material for: Airway environment drives the selection of quorum sensing mutants and promote Staphylococcus aureus chronic lifestyle
Source: Nat Commun. 2023 Dec 8;14:8135. doi: 10.1038/s41467-023-43863-2 (PMC10709412; doi:10.1038/s41467-023-43863-2)
Supplement: Supplementary file 6 — Reporting Summary [file 41467_2023_43863_MOESM6_ESM.pdf]

nature portfolio

Corresponding author(s): Anne Jamet

Last updated by author(s): 2023/11/08

Reporting Summary

Nature Portfolio wishes to improve the reproducibility of the work that we publish. This form provides structure for consistency and transparency in reporting. For further information on Nature Portfolio policies, see our Editorial Policies and the Editorial Policy Checklist.

Statistics

For all statistical analyses, confirm that the following items are present in the figure legend, table legend, main text, or Methods section.

n/a	Confirmed	
☐	☒	The exact sample size (*n*) for each experimental group/condition, given as a discrete number and unit of measurement
☐	☒	A statement on whether measurements were taken from distinct samples or whether the same sample was measured repeatedly
☐	☒	The statistical test(s) used AND whether they are one- or two-sided<br>*Only common tests should be described solely by name; describe more complex techniques in the Methods section.*
☒	☐	A description of all covariates tested
☐	☒	A description of any assumptions or corrections, such as tests of normality and adjustment for multiple comparisons
☐	☒	A full description of the statistical parameters including central tendency (e.g. means) or other basic estimates (e.g. regression coefficient) AND variation (e.g. standard deviation) or associated estimates of uncertainty (e.g. confidence intervals)
☐	☒	For null hypothesis testing, the test statistic (e.g. *F*, *t*, *r*) with confidence intervals, effect sizes, degrees of freedom and *P* value noted<br>*Give P values as exact values whenever suitable.*
☒	☐	For Bayesian analysis, information on the choice of priors and Markov chain Monte Carlo settings
☒	☐	For hierarchical and complex designs, identification of the appropriate level for tests and full reporting of outcomes
☒	☐	Estimates of effect sizes (e.g. Cohen's *d*, Pearson's *r*), indicating how they were calculated

Our web collection on statistics for biologists contains articles on many of the points above.

Software and code

Policy information about availability of computer code

Data collection	No software was used for data collection
Data analysis	RNA-seq analysis was based on STAR (v2.7.6a) and R package DESeq2 (v1.26.0). 16S rRNA sequences were analyzed using QIIME2—version 2019. Statistics were performed with Prism GraphPad software (8.02). Taxonomic classification was carried out by using Kraken2 and Kraken2 Standard Database after removal of human DNA with Hisat2. The Kraken2 reports were visualized as Sankey diagrams generated with Pavian. For the detection of sialidase genes, an assembly step (SPAdes v3.13.0 option --meta min_contig_coverage_threshold 1 and min_contig_length_threshold 1000) followed by an annotation step (RASTtk) were performed. Genomes were assembled with unicycler and annotated with prokka v1.11 to be used as references by snippy v3.1.

For manuscripts utilizing custom algorithms or software that are central to the research but not yet described in published literature, software must be made available to editors and reviewers. We strongly encourage code deposition in a community repository (e.g. GitHub). See the Nature Portfolio guidelines for submitting code & software for further information.

Data

Policy information about availability of data

All manuscripts must include a data availability statement. This statement should provide the following information, where applicable:

- Accession codes, unique identifiers, or web links for publicly available datasets
- A description of any restrictions on data availability
- For clinical datasets or third party data, please ensure that the statement adheres to our policy

The source data underlying all the main Figures and Supplementary Figures are provided as a Source Data file. Sequence data and RNA-seq data are available at https://doi.org/10.5281/zenodo.8380806 and https://www.ncbi.nlm.nih.gov/bioproject/PRJNA1036601. The datasets generated and analysed during this study are provided in Dataset_S1, Dataset_S2. Source data are provided with this paper.

Research involving human participants, their data, or biological material

Policy information about studies with human participants or human data. See also policy information about sex, gender (identity/presentation), and sexual orientation and race, ethnicity and racism.

Reporting on sex and gender	This study contains data on 9 cystic fibrosis patients, with no mention of sex/gender. According to the Cystic Fibrosis Foundation Patient Registry, the ratio of male to female patients is 51.8:48.2.
Reporting on race, ethnicity, or other socially relevant groupings	This study did not mention any race, ethnicity, or other socially relevant groupings.
Population characteristics	These 9 patients were diagnosed with cystic fibrosis with CFTR mutation F508del. The median age is 15 (range 11-17).
Recruitment	9 patients with cystic fibrosis were randomly selected by microbiology laboratory of Necker-Enfants Malades University Hospital, Paris, France.
Ethics oversight	The research procedure is validated by Ile-de-France IRB (French National Institute of Health and Medical Research, Ethics Review Committee). The study involving human research participants was approved by Assistance Publique–Hôpitaux de Paris (AP-HP).

Note that full information on the approval of the study protocol must also be provided in the manuscript.

Field-specific reporting

Please select the one below that is the best fit for your research. If you are not sure, read the appropriate sections before making your selection.

☒ Life sciences ☐ Behavioural & social sciences ☐ Ecological, evolutionary & environmental sciences

For a reference copy of the document with all sections, see nature.com/documents/nr-reporting-summary-flat.pdf

Life sciences study design

All studies must disclose on these points even when the disclosure is negative.

Sample size	Sample size was predetermined for mouse experiment based on previous experimental data (doi:10.1038/nm1079, DOI: 10.1038/s41467-021-21718-y). A total of 20 mice were divided into 4 groups. Other samples sizes were determined to reveal statistical significance and reproducibility of the data. For some analyses sample size was limited by availability of appropriate materials (analysis of patient material).
Data exclusions	No data were excluded from the analysis.
Replication	Experiments were performed at least in triplicate unless noted otherwise. All methods are described in the manuscript in order to allow replication of results. All attempts at replication were successful.
Randomization	Mice were randomly selected for experimental or control groups and kept in separate cages throughout the experiment. All the in vitro experiments each group was selected randomly.
Blinding	No formal blinding was used in this study. Because all experiments were performed based on standardized protocols and blinding has no effect on the experiment results. Unbiased experimental procedure and data analysis were carried out as far as possible.

Reporting for specific materials, systems and methods

We require information from authors about some types of materials, experimental systems and methods used in many studies. Here, indicate whether each material, system or method listed is relevant to your study. If you are not sure if a list item applies to your research, read the appropriate section before selecting a response.

Materials & experimental systems

n/a	Involved in the study
☒	☐ Antibodies
☐	☒ Eukaryotic cell lines
☒	☐ Palaeontology and archaeology
☐	☒ Animals and other organisms
☒	☐ Clinical data
☒	☐ Dual use research of concern
☒	☐ Plants

Methods

n/a	Involved in the study
☒	☐ ChIP-seq
☒	☐ Flow cytometry
☒	☐ MRI-based neuroimaging

Eukaryotic cell lines

Policy information about cell lines and Sex and Gender in Research

Cell line source(s)	ATCC (Calu-3) and University of California, San Francisco (CFBE41o-)
Authentication	Calu-3 cells obtained from ATCC were certified authentic, authenticity was monitored based on morphology. CFBE41o- were authentified by University of California, San Francisco.
Mycoplasma contamination	Calu-3 cells obtained from ATCC were certified mycoplasma free. CFBE41o- cell line was tested for mycoplasma contamination.
Commonly misidentified lines (See ICLAC register)	No commonly misidentified lines were used.

Animals and other research organisms

Policy information about studies involving animals; ARRIVE guidelines recommended for reporting animal research, and Sex and Gender in Research

Laboratory animals	7-week-old C57BL/6J female mice
Wild animals	This study did not involve wild animals.
Reporting on sex	Only female mice were used.
Field-collected samples	The study did not involve samples collected from the wild
Ethics oversight	Agreement from the ministry of higher education, research, and innovation of France.

Note that full information on the approval of the study protocol must also be provided in the manuscript.
